# Fully Adaptive Particle Filtering Algorithm for Damage Diagnosis and Prognosis

**DOI:** 10.3390/e20020100

**Published:** 2018-01-31

**Authors:** Elaheh Rabiei, Enrique Lopez Droguett, Mohammad Modarres

**Affiliations:** 1 Center for Risk and Reliability, Department of Mechanical Engineering, University of Maryland, College Park, MD 20742, USA; eld@umd.edu or; 2 B. John Garrick Institute for the Risk Sciences, University of California (UCLA), Los Angeles, CA 90095, USA; 3 Department of Mechanical Engineering, University of Chile, Santiago 8370448, Chile

**Keywords:** fully adaptive particle filtering, cross entropy method, relative entropy, Kullback–Leibler divergence, adaptive measurement model, diagnosis and prognosis, composite degradation

## Abstract

A fully adaptive particle filtering algorithm is proposed in this paper which is capable of updating both state process models and measurement models separately and simultaneously. The approach is a significant step toward more realistic online monitoring or tracking damage. The majority of the existing methods for Bayes filtering are based on predefined and fixed state process and measurement models. Simultaneous estimation of both state and model parameters has gained attention in recent literature. Some works have been done on updating the state process model. However, not many studies exist regarding an update of the measurement model. In most of the real-world applications, the correlation between measurements and the hidden state of damage is not defined in advance and, therefore, presuming an offline fixed measurement model is not promising. The proposed approach is based on optimizing relative entropy or Kullback–Leibler divergence through a particle filtering algorithm. The proposed algorithm is successfully applied to a case study of online fatigue damage estimation in composite materials.

## 1. Introduction

The particle filtering (PF) approach has received significant attention recently as a very powerful and flexible tool for damage diagnosis and prognosis. Its popularity has increased rapidly in the reliability field and various versions, such as auxiliary PF [1], regularized PF [2] and unscented PF [3], are proposed to improve the performance of the standard particle filtering algorithm. The standard PF algorithm and its variants have been implemented for diagnostics and prognostics in a wide range of applications, including life prediction of batteries and fuel cells [4,5,6,7], degradation assessment and prediction in gears and bearings [8,9,10], health monitoring and prognostics of gas turbines [11], machine tools [12], and pumps [13,14], and also, in damage estimation and prediction of composite materials [15,16,17,18]. More application examples can be found in a recent review paper on the PF algorithm by Jouin et al. [19]. Wang et al. [20] also presents a comprehensive survey on the remarkable achievements in PF methods for solving single-target and multiple-target tracking problems in the presence of false or missing data.

Although the focus of this study is on a PF method for nonlinear state-parameter estimation, it is noteworthy that due to challenges in this field, new algorithms and technologies are continuously being developed. The review by Li et al. [21] represents the cutting-edge advances in parametric recursive filtering, namely Gaussian Approximation (GA) and Gaussian Mixture (GM) filters, to tackle general nonlinear stochastic processes.

Particle filtering, like any other state-space model, is defined based on two elements: the state process model, which shows the progression of the hidden state of interest, $x_{k}$, through time, $P\left(x_{k}|x_{k-1}\right)$; and the measurement model, which presents the relationship between the observed variables, $y_{k}$, and the hidden states, $x_{k}$, at each time step, $P\left(y_{k}|x_{k}\right)$. The final goal is to estimate the posterior probability distribution of the hidden variable given all the observations, $P\left(x_{k}|y_{1:k}\right)$.

In recent studies, particular attention has been paid to developing and updating the state process model. Although several methods have been proposed and applied for simultaneous state-parameter estimations, online updating of both states and parameters concurrently is a long-lasting problem [22] and still needs improvement.

A common online Bayesian practice for simultaneous state-parameter estimation is augmenting the state vector to consider both states and parameters. The very first approach was to assign a prior distribution for the model parameters and then update states and parameters simultaneously as a unified filtering problem [23]. This approach, however, results in degeneracy of the parameter particles [22].

To overcome this issue, the addition of artificial noise to the model parameters in the augmented state vector was proposed [24,25]. It was realized very early that this approach resulted in more dispersed posterior distributions; therefore, methods based on kernel smoothing [23,26,27,28,29,30] were proposed to control the random walk of the parameters. There are also some limitations for this approach which are summarized in [30,31]. Tulsyan et al. [30] presents a comprehensive review on the current methods for simultaneous state-parameter estimation.

Despite all the advances in this field, in practice, most of the time, particular focus is only on updating the parameters of the state process model, while the parameters of measurement models are considered to be completely known.

The measurement model plays a very important role in updating both states and model parameters. In the literature, usually two types of measurements ($y_{k}$) and, consequently, two types of measurement models, are considered:

(1) The most common technique is that “$y_{k}$ is the same quantity as $x_{k}$”, which can be measured with some error. In other words, it is assumed that we can observe the hidden variable with some measurement error or uncertainty, expressed by $v_{k}$, which has two components of systematic and stochastic variability. Some examples can be found in [4,11,32,33,34]. This leads to a very basic and simple linear measurement model, as follows:
  (1) $$y_{k}=x_{k}+v_{k}$$
(2) The second group can be considered as the cases when “$y_{k}$ is NOT necessarily the same quantity as $x_{k}$”; for example in [16,35,36,37]. Therefore, $y_{k}$ has a different nature and needs to be related to the hidden variable through some physical or data-driven model:
  (2) $$y_{k}=h\left(x_{k},v_{k}\right)$$

An example for this case is when the hidden variable is the crack size and we observe acoustic emission or ultrasonic waves instead. This case is closer to real-world situations; however, the relationship between the hidden variable and observed variables is not always straightforward to develop.

Although one of the powerful features of PF is dealing with nonlinear measurement models, the majority of the published papers are based on case (1) in which the measurement model is a pure linear function that is simply constructed by adding noise to the hidden variable.

Also, in cases when the observed variable is different than the hidden state, i.e., case (2), usually a fixed, predefined measurement model has been used. However, in real-world applications, most of the time, no predefined function exists to exactly explain the relationship between the state of the system and the observed variable for that particular study. This is especially noteworthy for online monitoring, diagnostics and prognostics. On the other hand, any prior measurement model that might exist usually comes from previous similar experiments or related literature and does not necessarily fit the upcoming monitoring data for the particular case under study. The reason is that even identical components do not show the exact same behavior under equal operating/testing conditions.

Thus, there is a need to consider cases where not only the state process model, but also the exact measurement model, is not completely known. Therefore, the approach would be a significant step forward to real-time degradation monitoring and life prediction. In particular, it is useful for Structural Health Monitoring (SHM) in real-world applications because, when performing the damage monitoring and prognostics in real-time, both of the underlying state processes (degradation behavior) and measurement models (correlation between underlying degradation and condition-based monitoring data) are not fully defined in advance for the particular component/system under specific operating conditions.

The attempt for online estimation of the measurement model parameters, in addition to process model parameters, in the literature is to consider all the unknown model parameters in one augmented state vector and treat all of them in the same way [30,31,38,39]. However, this approach is questionable as parameters of state process models and measurement models are different in nature. This paper presents a new online approach for updating parameters of both process and measurement models separately but simultaneously.

The method is constructed on top of augmented PF, only for parameters of the state process model and adds the capability of adjusting the measurement model on the fly separately, based the concept of cross entropy as the foundation of the proposed fully adaptive particle filtering, in order to learn the parameters of the measurement model over time.

The rest of this paper is organized as follows: First, a brief introduction on augmented PF is presented in Section 2. Then, the idea of fully adaptive PF is proposed, and its mathematical details are discussed, in Section 3. Later on, Section 4 presents an application of the proposed method in a case study of damage estimation in a composite component under fatigue. The results are compared with augmented PF to demonstrate the capability of the proposed fully adaptive PF in dealing with close to real-time damage estimation. Finally, some possible application challenges are presented and discussed in Section 5.

## 2. Augmented Particle Filtering

The interest in extending the original PF to augmented PF was aroused recently, in order to estimate both the process model parameters and states simultaneously [23,26,27,40,41]. This is particularly useful when dealing with uncertain dynamic systems in which all or some of the process model parameters might be unknown. Therefore, in addition to estimating the evolution of damage state over time, it is necessary to learn the state process model by online tuning its parameters as well.

The extension of standard PF to augmented PF is not trivial. One of the conventionally proposed strategies [26] is to treat the model parameters the same way as states, which results in estimating the augmented state space problem as $P\left(x_{k},\theta_{k}|y_{1:k}\right)$. Therefore, the state process model considers parameter evolution as well:

(3) $$\begin{array}{c} \theta_{k}=g\left(\theta_{k-1},\gamma_{k-1}\right)\to p(\theta_{k}|\theta_{k-1})\\ x_{k}=f\left(x_{k-1},\theta_{k},\omega_{k}\right)\to p(x_{k}|x_{k-1},\theta_{k}) \end{array}$$

where g and γ are the transition function and random noise for model parameters (θ), respectively. Similarly, f and ω are the evolution function/model and random noise for states (x). Subscript *k*, refers to time step *k*. When measurements are available, both states and parameters should be updated using the predefined fixed measurement model.

The kernel smoothing method was proposed by [26] to control the additional noise introduced to a system via variance in model parameters. The idea of kernel smoothing is to first reduce the variability in parameters’ particles by shrinking them (with shrinkage parameter *a*) towards the current estimated mean ($\bar{\theta }$), and then to add controlled reduced noise ($a^{2}\gamma$) for the next step in the estimation process, as expressed in Equation (4). In this equation, *m* is the kernel location calculated for each particle (*i*):

(4) $$m_{k-1}^{i}=\left(\sqrt{1-a^{2}}\right)\theta_{k-1}^{i}+\left(1-\sqrt{1-a^{2}}\right){\bar{\theta }}_{k-1}$$

The value of *a* ∈ [0, 1] is suggested in [10,26] to be less than 0.2 for slowly varying particles and more than 0.8 for highly stochastic processes. Interested readers can find more details about the application of augmented PF in [15,36].

## 3. Proposed Fully Adaptive Particle Filtering Approach

In this section, the concept of fully adaptive PF is explained in more detail, and its features are compared with standard PF and augmented PF. Then, the underlying mathematics of the approach is presented and discussed.

The idea of fully adaptive particle filtering deals with the situation of the parameters of the measurement model being unknown, in addition to the state process model. Therefore, nothing is fixed or predefined in advance. This is a more complex problem because in order to update the states and process model parameters, a measurement model is required and, in the proposed fully adaptive PF approach, the measurement model itself is partially known and is adaptively changing, based on new observations.

Similar to the strategy for updating the state process model [26], it is supposed here that the measurement model is also “partially known”. That is, the “functional form” of the model is known, but not the value of its parameters. This requirement can be met in practice by generating an offline data-driven or a physics-based model, based on previous test data or relevant data from the literature. For example, suppose that the results of a set of fatigue experiments are available in which the fatigue crack length is the hidden variable of interest (*x*) and the acoustic emission signals are the observations (*y*). Thus, an a priori observation model can be built offline that correlates the acoustic emission signal to crack size, based on the historic experimental data. Now, if this model is to be applied in the PF algorithm for damage estimation in a new experiment with different loading conditions, the a priori fitted model needs to be updated online, to adjust to the conditions of the new experiment as measurement data comes in. More specifically, both the state process model and measurement model are condition-based and need to be adjusted to the particular case under study.

### 3.1. Proposed Measurement Adaptation Approach

The proposed approach for adaptive measurement is based on minimizing the relative entropy, also known as Kullback–Leibler divergence, or KL-divergence, or KLD for short [42,43]. Relative entropy between two probability distributions on a random variable is a measure of the distance between them. More formally, relative entropy or KLD is an effective evaluation method to compare two distributions, P and Q, as:

(5) $$D_{KL}(P||Q)=\int P\left(x\right)\log\frac{P\left(x\right)}{Q\left(x\right)}dx$$

Usually, P is the true distribution of the data (the objective distribution) and Q comes from the model used to approximate that true distribution.

To apply the idea of KLD to the adaptive measurement model, P is the distribution of the real measurement that is captured online at each time step (*k*) with some degree of uncertainty. On the other hand, Q is the distribution built based on predicted measurements at that time, coming from the previously-updated measurement model. In standard and augmented PF, predicted measurements are calculated by applying the fixed and predefined measurement models on the particles after they are propagated using the state process model. Here, predicted measurements need to be calculated using a partially known (approximate) measurement model ($\tilde{h}$). Since the exact values of the parameters in $\tilde{h}$ are unknown, the measurement model might be inaccurate, which will lead to false predicted measurements. In this situation, the distribution (Q), fitted to the predicted observations, might be significantly different than the distribution of the real observation (P). The main idea is to optimize the parameters in the measurement model ($\tilde{h}$) with the objective of minimizing the KLD between Q and P.

This optimization problem is applied through the cross entropy method (CE method), which is rooted in the adaptive variance minimization algorithm for the rare event probability estimation in stochastic networks [44,45]. In general, the CE method is performed in two iterative phases [46]:

(1) (Generation of a set of random samples (e.g., vectors, trajectories) according to a specified parameterized model.
(2) Updating of the model parameters, based on data generated in the previous step, to produce “better” samples in the next iteration. This is done by KLD minimization.

To better understand the CE method, let $X=\left(X_{1},\ldots ,X_{n}\right)$ be a random vector with a probability density function f(X;v) with respect to parameter v. Consider S to be some real-valued function on X. Suppose the goal is to find the minimum of S over X and the corresponding minimizer, $x^{*}$:

(6) $$S\left(x^{*}\right)=\gamma^{*}=\min\left(S\left(x\right)\right)$$

Accordingly, the rare-event probability (ℓ) can be defined as the probability that S(X) is equal or less than some real value (γ) as ℓ=P(S(X)≤γ). This probability would be very small (i.e., rare event), if the level (γ) is chosen to be very close to $\gamma^{*}$. One of the most effective ways of estimating rare-event probabilities is to use importance sampling. Therefore, the importance sampling estimator of ℓ can be defined as:

(7) $$\hat{\ell }=\frac{1}{N}\overset{N}{\underset{i=1}{\sum }}\frac{f\left(X_{i},v\right)}{g\left(Xi\right)}I_{\left\{S\left(X_{i}\right)\leq \gamma \right\}}$$

In which, $I_{\left\{S\left(X_{i}\right)\leq \gamma \right\}}$ is the indicator function that gets a value of 1 when the condition $\left\{S\left(X_{i}\right)\leq \gamma \right\}$ is met and 0 otherwise. g(X) is the importance sampling density function and $\left(X_{1},\ldots ,X_{N}\right)$ are iid samples drawn from g(X). The optimal importance sampling density that provides zero variance can be defined by the change of measure:

(8) $$g^{*}\left(Xi\right)\equiv \frac{f\left(X_{i},v\right)I_{\left\{S\left(X_{i}\right)\leq \gamma \right\}}}{\ell }$$

However, $g^{*}\left(Xi\right)$ depends on the unknown value (ℓ). Therefore, the idea of the CE method is to choose the parameter vector in $f\left(X_{i},v\right)$, such that the distance between the densities, $g^{*}\left(X\right)$ and f(X;v), is minimal. This can be done by minimizing the KLD:

(9) $$D_{KL}\left(g^{*}\left(x\right)||f\left(x;v\right)\right)=\int g^{*}\left(x\right)\log g^{*}\left(x\right)dx-\int g^{*}\left(x\right)\log f\left(x;v\right)dx$$

Minimizing $D_{KL}\left(g^{*}||f\left(x;v\right)\right)$ is equivalent to maximizing the last term in Equation (9):

(10) $$\max\int g^{*}\left(x\right)\log f\left(x;v\right)dx=\max\int \frac{f\left(x,v\right)I_{\left\{S\left(x\right)\leq \gamma \right\}}}{\ell }\log f\left(x;v\right)dx$$

which can be estimated with another round of importance sampling. More details can be found in [45,46,47].

In practice, in order to apply the CE method, the sampling distribution is not required to be related to the objective function (S) and can be chosen quite arbitrarily. Usually, Gaussian distributions with independent means, $\mu_{t}={\left(\mu_{1},\mu_{2},\ldots ,\mu_{n}\right)}_{t}$, and standard deviations, $\sigma_{t}={\left(\sigma_{1},\sigma_{2},\ldots ,\sigma_{n}\right)}_{t},$ are used to generate random samples for each of the unknown variables, $X=\left(X_{1},\ldots ,X_{n}\right)$. At each iteration of the CE method, vectors of $\mu_{t}$ and $\sigma_{t}$ will be updated. And at the end of the algorithm with a predefined stopping threshold, sequences of $\mu_{t}$ and $\sigma_{t}$ are generated in a way that the vector of the mean of the parameters ($\mu_{t}$) tends to the optimizer ($X^{*}$), and the vector of standard deviations ($\sigma_{t}$) tends to the zero vector.

Recently, KLD was introduced to the PF algorithm as a statistical approach in the context of mobile robot localization [48,49], to increase the efficiency of particle filters by “adapting the sample size” during the estimation process. In another study [30], KLD was implemented in the augmented PF, but, it was only used to tune/optimize the kernel width. The proposed approach in our research, however, implements the concept of using KLD to update the whole measurement model and adjusting its parameters online, with a fixed number of particles. In the following text, we will determine how KLD is used to dynamically adapt the measurement model in PF over time.

### 3.2. Introducing the KLD into the Augmented Particle Filtering Algorithm

Consider the procedure of the augmented PF algorithm in which the state process model with unknown model parameters can be demonstrated by Equation (3). In the proposed fully adaptive PF algorithm, it is also assumed that the measurement model is partially defined. Suppose the analyst’s belief about the approximate prior measurement model is presented by:

(11) $${\tilde{y}}_{k}=\tilde{h}\left(x_{k},\varphi_{k},\tau_{k}\right)\to P({\tilde{y}}_{k}|x_{k-1},\varphi_{k})$$

where, φ is the measurement model parameters that need to be updated through time as new real-time monitoring data becomes available. τ is the uncertainty in the model ($\tilde{h}$), based on parameters and measurement uncertainty.

The proposed procedure of the fully adaptive PF is as follows:

At each time step (*k*), the state process model in Equation (3) is used to propagate the particles one step ahead in time. In standard or augmented PF, the predefined measurement model would be applied at this stage, to estimate the weights of the particles regarding the true observation at that time. However, since here, the measurement model is not fully defined in advance, before assigning the weights to the particles, the measurement model needs to be adjusted. Therefore, the tentative prior measurement model in Equation (11) will be used to generate a set of predicted observations ${\tilde{y}}_{k}$ based on the current particle state. Distribution $Q\left({\tilde{y}}_{k}^{i}|x_{k}^{i}\right),i=1,\ldots ,n$ is the probability distribution function fitted to the set of predicted observations, ${\tilde{y}}_{k}^{i}$.

On the other hand, when the real measurement ($y_{k}$) is captured with some known measurement noise (due to measuring instruments and procedure), $P(y_{k}|x_{k})$ can be defined as the probability distribution fitted to the real measurement ($y_{k}$) at time step *k*. Therefore, there would be two distributions at each time step: $P(y_{k}|x_{k})$ and $Q\left({\tilde{y}}_{k}^{i}|x_{k}^{i}\right)$, which ideally should be very similar. However, since the model parameters (φ) in $\tilde{h}$ are unknown, predicted measurements might be far from the real observation at time *k*. This will result in assigning negligible weights to the particles and, consequently, the algorithm will collapse because of the very low likelihood. However, the root of this problem is the fact that the measurement model is not completely defined in the first place. Therefore, to address this issue, the proposed approach is to update the measurement model by adjusting its parameters (φ) in a way that the distance between $Q\left({\tilde{y}}_{k}^{i}|x_{k}^{i}\right)$ and $P(y_{k}|x_{k})$ is minimized. Using the KLD in Equation (5), the optimum φ can be found based on real measurement data at time *k*. Then, the updated measurement model is used to calculate the weights of the particles as before. This procedure will be repeated every step as a new measurement is captured. Algorithm 1 presents the algorithm for the fully adaptive PF, which is based on augmented PF with kernel smoothing.

**Algorithm 1.** Proposed fully adaptive PF algorithmInitiation step: Sample *N* particles from initial distributions of states and parameters: $x_{0}^{i}\sim p\left(x_{0}\right)\;i=1,2,\ldots ,N$
$$\theta_{0}^{i}\sim p\left(\theta_{0}\right)\;i=1,2,\ldots ,N$$ Assign initial equal weights to all the particles$$w_{0}^{i}\sim \frac{1}{N}\;i=1,2,\ldots ,N$$ Recursive steps: *Prediction:* Estimate $m_{k-1}^{i}$ for each parameter using the shrinkage rule in Equation (4) Draw new samples for parameter vector from:$$\theta_{k}^{i}\sim N\left(\cdot |m_{k-1}^{i},a^{2}\gamma_{k-1}\right)$$ Propagate each particle one step forward using state process model with new sampled parameter:$$x_{k}^{i}\sim p(\cdot |x_{k-1}^{i},\theta_{k}^{i})$$ *Update:* Compute the predicted observations using the tentative measurement model: $${\tilde{y}}_{k}=\tilde{h}\left(x_{k},\varphi_{k},\tau_{k}\right)$$ Update the parameters of $\tilde{h}$ to minimize the KLD:
$$\varphi_{k}=\arg\min\left\{D_{KL}\left(P||Q\right)\right\}=\arg\min\left\{\int P\left(y_{k}|x_{k}^{i},\theta_{k}^{i}\right)\log\left[\frac{P(y_{k}|x_{k}^{i},\theta_{k}^{i})}{Q\left({\tilde{y}}_{k}^{i}|x_{k}^{i},\theta_{k}^{i},\varphi_{k}\right)}\right]\right\}$$ Calculate the weights for each particle as new measurement ($y_{k}$) becomes available:
$$w_{k}^{i}=w_{k-1}^{i}\cdot p\left(y_{k}|x_{k}^{i},\theta_{k}^{i},\varphi_{k}\right)$$ Normalize the weights:$$w_{k}^{i}=\frac{w_{k}^{i}}{\sum_{i=1}^{N}w_{k}^{i}}$$ *Estimate:* Estimate the expected state:$${\bar{x}}_{k}=\overset{N}{\underset{i=1}{\sum }}w_{k}^{i}\cdot x_{k}^{i}$$ *Resample:* Resample (with replacement) new set of particles for states and process model parameters ${\left\{x_{k}^{i},\theta_{k}^{i}\right\}}_{i=1}^{N}$ based on calculated weights ($w_{k}^{i}$).

## 4. Case Study: Real-Time Damage Estimation in Composite Material

The use of composite materials in industry is increasing rapidly because of their low weights, high strengths and long fatigue lives. However, the inhomogeneity of composite materials makes the degradation process very complicated, especially when subjected to cyclic loading. Consequently, predicting damage growth and estimating the remaining useful life in real-time is a challenging endeavor.

In this section of the paper, the capability of the proposed fully adaptive PF to handle the real-world situations is presented when both state process and measurement models are not fully known and need to be adjusted for the particular case under study.

Accordingly, the published results of Naderi, et al.’s experiment [50] on Glass/Epoxy (G10/FR4) composite laminate during bending fatigue damage are used to demonstrate fully adaptive PF. Indeed, Reference [50] presented the dissipated thermal energy evolution versus the number of cycles for two fatigue experiments, both subjected to a frequency of 10 Hz, but with different displacement amplitudes of (a) 46.54 mm and (b) 38.1 mm, respectively. The total number of cycles at failure for experiments (a) and (b) were 4000 and 6800, respectively. The variation in the component’s surface temperature during the degradation was also monitored and reported for both test conditions.

Damage is defined as the normalized dissipated thermal energy, $D=\frac{H}{H_{f}}$, where $H_{f}$ is the final value of the dissipated energy at the time when failure occurs. $H_{f}$ is estimated to be approximately 1.43 times the value of dissipated energy measured at about 85% of the total fatigue life in a tension–tension test [50].

Damage evolution through time (n) is estimated using $D=1-{\left(1-{\left(\frac{n}{N_{f}}\right)}^{B}\right)}^{A}$, in which $N_{f}$ is the total life and A and B are model parameters. In order to convert it to the form of the state process model, one can discretize this damage evolution model with sufficiently small ΔN as:

(12) $$D_{k}=D_{k-1}+\frac{\Delta D}{\Delta n}{|}_{k-1}\cdot \Delta n\cdot e^{\omega_{k}}$$

where, *ΔD*/*Δn* is the derivative of *D* with respect to n. $e^{\omega_{k}}$ is used to show the random behavior of the state process model; *ω* is white Gaussian noise with mean zero and standard deviation σ, and *k* is the count of fatigue load cycle.

The temperature measurements induced by thermal dissipation around the fatigue damage area and detected by an infrared device, are considered to be the monitoring data for updating the damage estimation via the measurement model:

(13) $$T_{k}=\varphi_{0}+\varphi_{1}\cdot D_{k}+\varphi_{2}\cdot {D_{k}}^{2}+\varphi_{3}\cdot {D_{k}}^{3}+\upsilon_{k}$$

Interested readers can refer to [15] for more detail on this case study and how to set up the problem in the frame of augmented PF.

**Figure 1:**
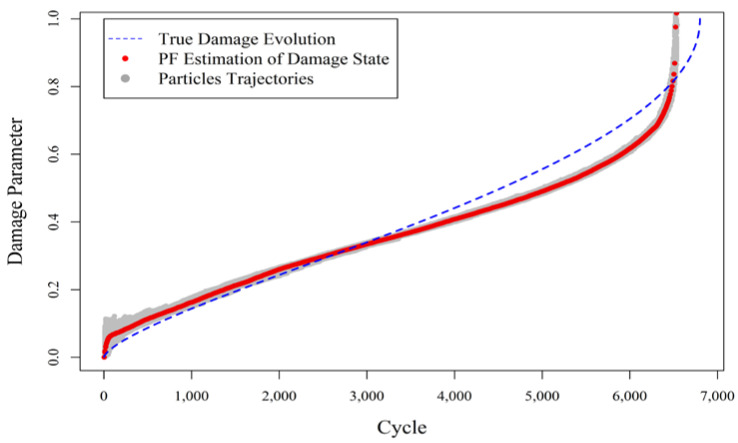
Damage estimation in a composite specimen under fatigue loading (experiment (b)), using augmented particle filtering (PF) with the fixed, but uncertain predefined measurement model coming from experiment (a).

Now, consider the scenario in which test (a) has already been performed, and the results are available. Suppose the developed state process and measurement models based on test (a) are, respectively, the only available information about the evolution of damage and its possible relationship to the online monitoring data. Then, this information is going to be applied online, to estimate and predict real-time damage in the second experiment that is subjected to different test conditions. The augmented PF provides the capability of updating the parameters of the state process model to adjust it to the new condition in test (b). However, it cannot update the measurement model. Figure 1 shows the results of the fatigue damage estimation when the predefined measurement model with fixed model parameters obtained from the first experiment is used in the augmented PF for damage estimation in experiment (b).

As expected and demonstrated in Figure 1, the augmented PF substantially deviates from the true track of the damage evolution in the component, because the existing measurement model was derived from a different (albeit related) experiment and was not adjusted for the particular damage progression at hand. This underlines the importance of learning/updating the measurement model in addition to the state process model.

Therefore, in real-time SHM, it is necessary to adaptively update the parameters of the measurement model encapsulated in $\varphi_{k}$. For this case study, $\varphi_{k}$ is as follows:

(14) $$\varphi_{k}={\left[\varphi_{0},\varphi_{1},\varphi_{2},\varphi_{3}\right]}_{k}$$

The state process model is the same as before, and is given by Equation (3). Thus, the process model parameter vector is $\theta_{k}={\left[A,B,N_{f}\right]}_{k}$. Note that the total life of component $N_{f}$ is unknown and therefore, is treated as another model parameter that needs to be learned.

**Figure 2:**
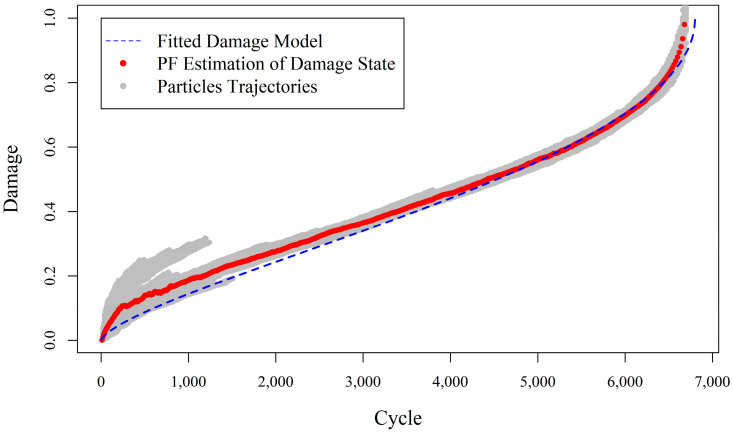
Damage estimation in a composite specimen under fatigue loading (experiment (b)) using fully adaptive PF which updates the parameters of the inaccurate pre-existing measurement model coming from experiment (a).

**Figure 3:**
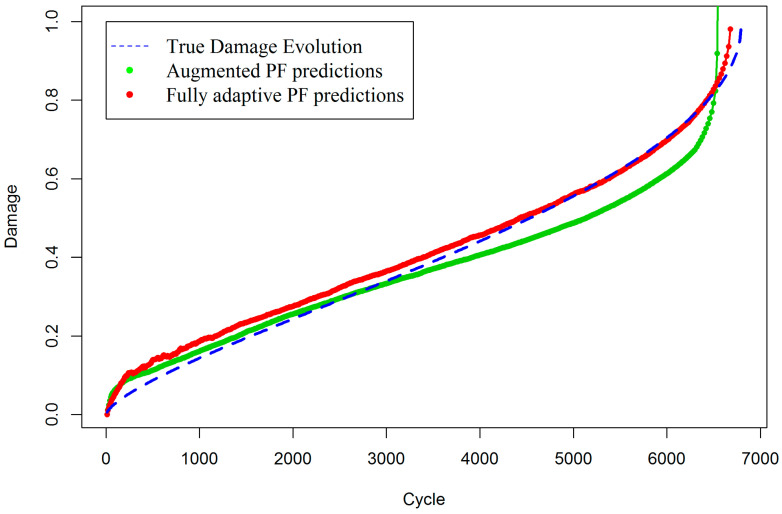
Comparison between augmented PF with the fixed measurement model and fully adaptive PF.

Figure 2 shows that the proposed fully adaptive PF approach can drastically improve the estimation of true damage in the composite when both state process and measurement models are partially known. A comparison of Figure 1 and Figure 2 reveals the strength and improvements in the proposed fully adaptive PF, in situations where the pre-existing measurement model involves a high level of uncertainty. For better demonstration, Figure 3 presents damage estimations of the two methods, side by side. In fact, in the early stages of damage evolution, the predictions of fully adaptive PF are not as good as augmented PF, with the predefined measurement model. This is mainly because fully adaptive PF starts the estimations without any accurate knowledge about both state and measurement model parameters. Therefore, it is expected to provide less accurate, but still reasonable, results at the beginning. Indeed, the closer the initial values of the parameters to their expected true values, the faster and more robust convergence will be achieved. Here, it is intended to show that even when the analyst does not have enough information about the true values of the model parameters, the fully adaptive PF is still capable of estimating both states and parameters with reasonable accuracy. It can be seen that as time goes by and more measurement data arrives, the fully adaptive PF estimates the true damage evolution with less error, while the augmented PF significantly deviates from the true track of the damage, especially toward the end of life, which is usually a primary objective in SHM.

In order to mathematically show the increase in accuracy with the fully adaptive PF approach, the Root Mean Square Error (RMSE) is also calculated for both methods. The RMSE for the augmented PF is 0.057 and for fully adaptive PF is 0.022. Note that the last 200 cycles are not considered in the numerical calculation of RMSE, because in that period, the augmented PF completely diverges and produces infinite numbers, so the RMSE for augmented PF would be infinite.

**Figure 4:**
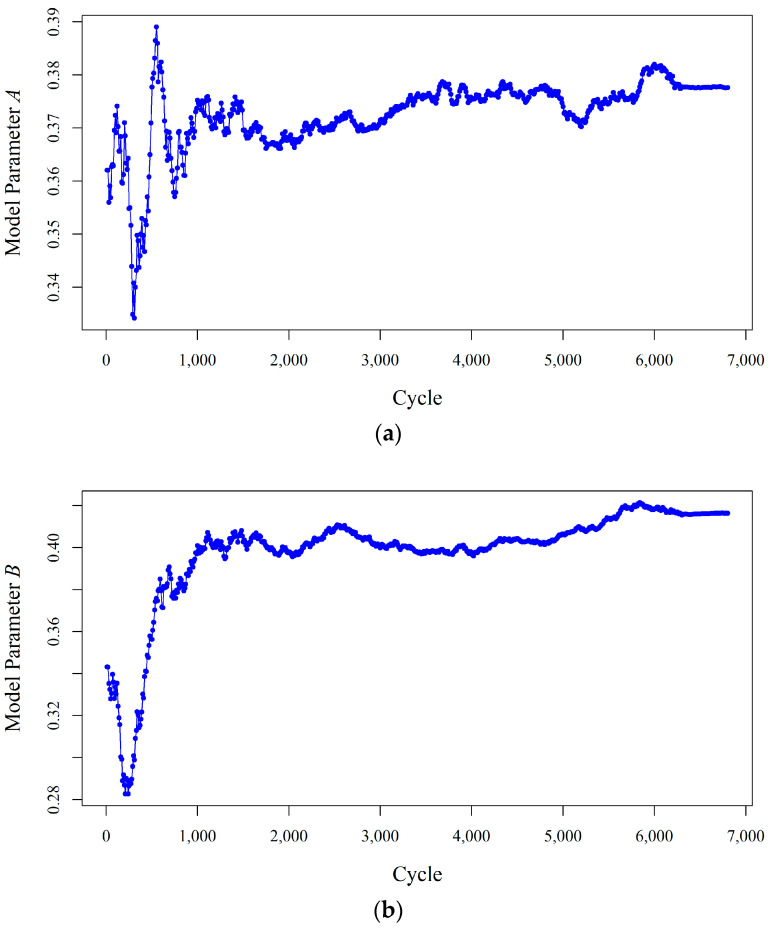

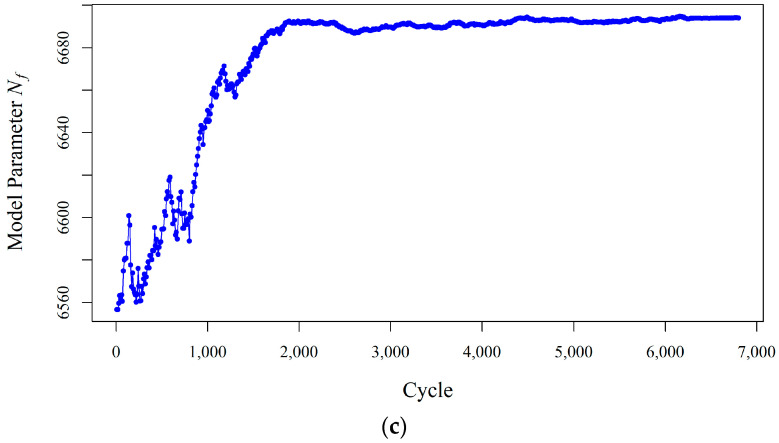
Updating the parameters of the state process model in the fully adaptive particle: (**a**) Model Parameter *A*; (**b**) Model Parameter *B*; (**c**) Model Parameter *Nf*.

Figure 4 shows how the state process model parameters change over time until their variations decrease in fully adaptive PF. A high level of uncertainty is observed (in both Figure 2 and Figure 4) at the beginning of the process because all the unknown model parameters are selected randomly. This uncertainty decreases through time when more measurements are obtained.

**Figure 5:**
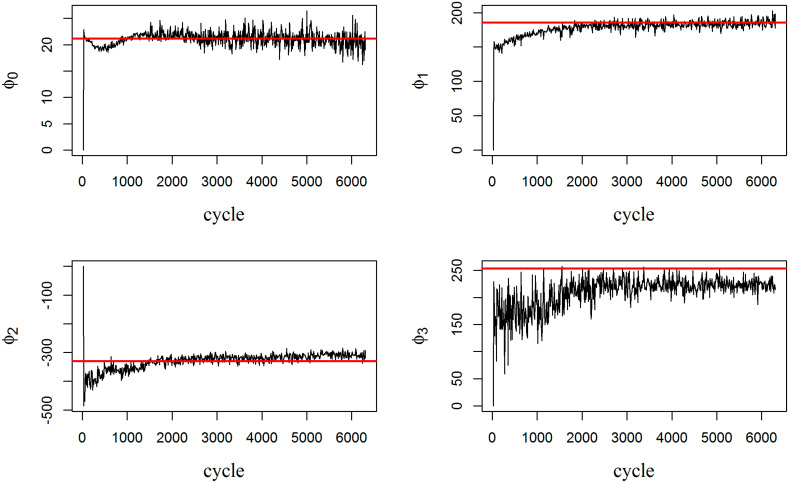
Parameters of the measurement model in fully adaptive PF.

Figure 5 tracks the convergence of measurement model parameters $\left[\varphi_{0},\varphi_{1},\varphi_{2},\varphi_{3}\right]$. The red line in each graph is the expected value for that parameter, coming from curve fitting. The expected values are derived offline, after completion of the experiment, by fitting the measurement model (Equation (13)) to all the recorded measurement data. Note that even though the parameters in the measurement model are noisy and do not necessarily converge to their expected values (especially, parameter $\varphi_{3}$), the combination of them is able to successfully estimate the damage, as presented earlier in Figure 2. Moreover, it is worth noting that the expected values of the parameters were estimated offline, from the available data. Thus, if the data is limited or scarce, the estimated expectations will be off the true population expectations for these parameters. Indeed, updating both the state process model and measurement model through fully adaptive PF delivers improved flexibility in tracking the true damage evolution.

All the computations in this research were performed in R programming language (The R Project for Statistical Computing) [51]. R codes were developed for augmented PF and fully adaptive PF and results were compared. Package CEoptim [47] was applied to carry out the cross entropy calculations in the proposed algorithm for fully adaptive PF.

## 5. Challenges of the Proposed Approach

The main concern in proposed fully adaptive PF is setting the initial values for model parameters, φ and θ. If the state process model has m_1_ parameters and the measurement model consists of m_2_ parameters, θ and φ will be vectors of sizes m_1_ and m_2_, respectively. Also, each of the model parameters is a random variable and needs the mean and standard deviation to be defined. Therefore, this will result in $2\times \left(m_{1}+m_{2}\right)$ unknown variables. In addition, there is the process noise (ω) to represent the stochasticity of the process, and the measurement noise (ν), which is related to the uncertainty in the measured data. Therefore, there are $2\times \left(m_{1}+m_{2}\right)+2$ random variables (hyper-parameters) in total that should be addressed accordingly.

The existence of multiple uncertain random variables results in additional uncertainty in the SHM. However, the proposed approach is mainly recommended for application in online monitoring contexts, where a high sampling rate (resulting in large-scale data sets) and continuous streaming of monitoring data are expected. More frequent observation data will increase the convergence rate in the model parameters and, therefore, the concern of uncertain estimations can be managed.

On the other hand, it might be argued that the method introduces some bias toward the measurement. In other words, minimizing the KLD can be considered to shift the probability distribution (Q) towards P, before weighing the particles. Therefore, the approach is sensitive to the true measurements. Indeed, this is a favorable property that makes sure the tentative inaccurate measurement model gets updated, based on real-time true measurements, before being used to weigh the particles.

However, if the arrival of real-time measurements is sparse, or too noisy, or not informative enough, there is a possibility that the method will fail or become skewed towards unreliable measurements. To overcome this problem, it can be beneficial to use a regularization parameter, to control how much the distribution (Q) is allowed to shift towards P, which is regularizing the KLD. Consequently, this method will prevent the distribution (Q) from getting too close to the distribution (P), if the measurement is not reliable enough. This idea needs to be validated in future studies.

Finally, the trade-off of dynamically updating the parameters of both the process model and measurement model, along with estimating the states, would clearly be more expensive computations. The approach requires solving an optimization problem at each time step, which comes at a high computational cost. For example, in the case study presented in Section 4, each time step for performing the augmented PF (with 1000 particles for each variable) was about 0.01 s on the personal computer (Dell, MD, USA) with 3.6 GHz Intel(R) Xeon(R) CPU, whereas, it took 0.02 to 0.21 s for every time step for fully adaptive PF to update both state process and measurement models.

## 6. Conclusions

In this paper, an algorithm for fully adaptive PF was proposed. The aim of the proposed approach was to develop a PF technique that requires neither the fully-known state process model, nor a predefined measurement model. Thus, both state process and measurement models are learned and updated online over time. It is particularly useful for performing fully-online, structural health monitoring. However, the approach is general and can be used for any tracking/monitoring/predicting purposes in real time, when a substantial amount of online monitoring data is available. The proposed algorithm incorporates the concept of KLD to update the parameters of the measurement model, based on real-time upcoming measurements, while the parameters of the process model are learnt via augmented PF as before. Also, potential concerns in applying the approach, including the existence of multiple uncertain hyper-parameters, bias toward upcoming measurements and computational costs, were discussed and possible solutions were proposed, which should be examined in future studies.
